# Towards Multidimensional Radiotherapy: Key Challenges for Treatment Individualisation

**DOI:** 10.1155/2015/934380

**Published:** 2015-03-05

**Authors:** Iuliana Toma-Dasu, Alexandru Dasu

**Affiliations:** ^1^Medical Radiation Physics, Stockholm University and Karolinska Institutet, P.O. Box 260, 171 76 Stockholm, Sweden; ^2^Department of Radiation Physics and Department of Medical and Health Sciences, Linköping University, 581 83 Linköping, Sweden

## Abstract

Functional and molecular imaging of tumours have offered the possibility of redefining the target in cancer therapy and individualising the treatment with a multidimensional approach that aims to target the adverse processes known to impact negatively upon treatment result. Following the first theoretical attempts to include imaging information into treatment planning, it became clear that the biological features of interest for targeting exhibit considerable heterogeneity with respect to magnitude, spatial, and temporal distribution, both within one patient and between patients, which require more advanced solutions for the way the treatment is planned and adapted. Combining multiparameter information from imaging with predictive information from biopsies and molecular analyses as well as in treatment monitoring of tumour responsiveness appears to be the key approach to maximise the individualisation of treatment. This review paper aims to discuss some of the key challenges for incorporating into treatment planning and optimisation the radiobiological features of the tumour derived from pretreatment PET imaging of tumour metabolism, proliferation, and hypoxia and combining them with intreatment monitoring of responsiveness and other predictive factors with the ultimate aim of individualising the treatment towards the maximisation of response.

## 1. Introduction

The progress and technological development of functional and molecular techniques for imaging tumours have offered the possibility of redefining the target in radiation therapy and devising the treatment in an innovative manner. Fourteen years have passed since Ling et al. [1] introduced the concept of biological target volume (BTV) encompassing the multidimensional physiological and functional information provided by the new imaging techniques. At the end of their seminal paper on multidimensional radiotherapy, the authors challenged the research and clinical communities to define a biological target volume and apply it by the year 2010 moving towards evidence-based multidimensional radiation therapy by conforming the physical dose distribution to the radiobiological features of the target that may be derived from molecular and functional imaging. Research has been conducted towards this aim, but the current practice in radiation therapy is still, at its best, based on the physical optimisation of the dose distribution according to the anatomical information regarding the localisation and the extent of the tumour and the normal tissue. The routine planning in clinical radiation treatment does not generally take into account the particular radiation sensitivity of the tumour of an individual patient or the spatial and temporal heterogeneity of tumour resistance. However, it is well known that these aspects may be the causes for treatment failure for a considerable fraction of the nonresponding patients, as the standard dose prescription does not ensure sufficiently curative doses to counteract the radiation resistance of the tumour. Among these adverse factors one has to count the variations in cellular density from tumour to tumour, the proliferation characteristics of the tumour cells, and the distinct microenvironmental characteristics of the tumours.

A broad array of techniques could now be used to determine the morphologic, functional, and molecular features of tumours and normal tissues. Among these, positron emission tomography (PET) has the advantage of being almost noninvasive as it uses tracers that are usually metabolic substitutes and is quite sensitive since quite low concentrations of tracers are required for imaging. Furthermore, several tracers are already available for investigating various processes [2]. The most quoted tumour phenotypes that could be integrated with the CT-defined gross tumour volume (GTV) to obtain the BTV are the tumour metabolism, proliferation, hypoxia, and angiogenesis. Consequently, several methods have been proposed for providing the relevant biological information on metabolic, biochemical, and physiological factors resulting in a new approach for the way the treatment is planned [3, 4]. However, it became clear that tumours do not contain one biological target volume to which a homogeneous dose could be prescribed, but instead the biological features of interest for targeting exhibit considerable heterogeneity with respect to magnitude, spatial, and temporal distribution, both within one patient and between patients.

This review paper aims to add to these conceptual solutions by discussing some of the key challenges for incorporating the radiobiological features of the tumour into the models for predicting the treatment outcome or for counteracting the adverse tumour control factors based on PET imaging of tumour metabolism, proliferation, and hypoxia.

## 2. PET Imaging of Key Tumour Phenotypes and Corresponding Models for Dose Painting

Several modelling studies have presented various approaches with different degrees of complexity to include the imaging information into treatment planning. Although in some cases more than one type of PET image was available, to the best of our knowledge the models currently proposed in the literature did not focus on more than one of the adverse tumour control factors at a time for determining the BTV and its subregions and for addressing the radiation resistance associated with them. With very large extent, the majority of the models focused on tumour hypoxia since it is one of the most important factors that determine the response of the tumour to radiation therapy [5–8]. A common approach is to delineate a hypoxic subtarget in the tumour based on PET tracers specifically designed for imaging the tumour oxygenation such as [^18^F]Fluoromisonidazole ([^18^F]FMISO), [^18^F]Fluoroetanidazole ([^18^F]FETA), [^18^F]Fluoroazomycinarabinofuranoside ([^18^F]FAZA), or the nonimidazoles compound Cu(II)-diacetyl-bis-N-(4)-methylthiosemicarbazone (Cu-ATSM) and prescribe an escalation of the dose according to available radiation therapy techniques and considering the tolerance of the normal tissues around the tumour [9–11]. However the risk of using such an empirical approach is that the prescribed dose might not be large enough to counteract the hypoxic radiation resistance and therefore the method might fail to bring the expected results in clinical settings. Other approaches recommended highly heterogeneous dose distributions based on a linear increase of the prescribed dose according to the signal intensity in the PET image [12, 13] or, as a result of redistributing the dose to the target, by increasing the dose to the hypoxic voxels while decreasing the prescribed dose to the remaining voxels in the tumour [14, 15]. More complex approaches for heterogeneous dose prescription make use of dynamic PET information [16]. However, heterogeneous dose distributions are at risk of failing to provide the expected results for cases of dynamic hypoxia as have indeed been seen in clinical patients [17] since changes in the spatial distribution of hypoxia could easily lead to mismatches between the hypoxic subregions and the planned hotspots in the dose distribution. Alternative approaches in which the impact of local changes in the oxygenation of the tumour were also theoretically explored and subsequently tested for feasibility [18, 19].

Another factor that reduces treatment effectiveness among the physiological factors that influence the response to treatment is tumour cell proliferation. Cell proliferation and especially accelerated repopulation that is seen in rapidly growing tumours like head-and-neck carcinomas are regarded as adverse factors for the success of radiotherapy because they increase the population of cells that could regrow the tumour and therefore need to be sterilised with radiation [20]. This warrants the noninvasive investigation of tumour proliferation as a potential target to increase local control. Proliferation and/or metabolic PET tracers may be used to image and quantify the tumour regions with increased proliferation. They can also be used to predict and evaluate the response to treatment and if necessary to adapt or improve the therapy.

Many tumours also have an increased glycolytic metabolism compared to normal tissues [21]. This means that a radioactive analogue of glucose will be easily taken up in tumours and the tumour burden could be detected through the difference in activity concentration. This is the case of [^18^F]Fluorodeoxyglucose ([^18^F]FDG) that led to PET being largely used for improving the detection and staging of cancers as well as for target delineation and the evaluation of treatment [22–24]. Other tracers, like ^11^C-acetate, would have to be considered for non-FDG-avid tumours, offering yet another facet of tumour metabolism [25–28].

Furthermore, some studies showed that the uptake of FDG in slowly proliferating tumours is generally lower than in rapidly growing, poorly differentiated tumours [2], suggesting an indirect correlation with the proliferating potential of the tumours. However, FDG is not a dedicated tracer for tumour proliferation and therefore some regions with increased glycolytic metabolism such as inflammations will also show a high FDG uptake and will be imaged, decreasing the quality of the information provided by FDG-PET. Due to these limitations other biomarkers have been proposed for specifically characterising tumour proliferation [2], such as [^18^F]3′-deoxy-3′-fluorotymidine ([^18^F]FLT). In comparison to hypoxia, little attention has been paid to developing models for the inclusion of proliferation information into treatment planning. To the best of our knowledge, there is only one study in which the possibility of including quantitative imaging of tumour proliferation and cell density into the radiobiological evaluation and optimisation of treatment planning was theoretically explored [29].

## 3. Multitracer Spatial and Temporal Distributions and TCP Models

The most common hypotheses on which a dose painting approach is generally based are the following: local recurrence is related to resistant foci not eradicated by the currently prescribed and delivered uniform doses, noninvasive functional and molecular imaging allows mapping the target in terms of radiation resistance and progress in treatment planning and radiation delivery allows nonhomogeneous target irradiation while the irradiation of the normal tissue and organs-at-risk (OARs) is kept below the tolerance levels. The subsequent steps that are taken when proposing a strategy for dose painting are to determine what functional noninvasive methods can be used for imaging specific tumour phenotypes related to local control or risk of relapse after (chemo)radiotherapy, to determine the response function(s) that would allow the quantitative interpretation of the image or images translated into a painting strategy, and finally to determine how to prescribe and deliver the dose, as dose boosting or as in the manner generally known as dose painting by numbers.

The larger variability in the known approaches for performing treatment planning based on functional imaging is related to the interpretation of the images and their translation into dose prescription. Furthermore, for the case of combined information regarding the key factors that should be accounted for when attempting dose painting based on functional PET imaging, tumour metabolism, proliferation, and hypoxia, the complexity of the problem has prevented up to the present date the proposal of quantitative radiobiological models to simultaneously account for them.

The most general approach for modelling the tumour response if information about the key features regarding tumour resistance to radiation is available as derived from functional imaging is to calculate the tumour control probability (TCP) at voxel level and then to integrate the response over the whole target structure. Thus, assuming an initial distribution of cell density *n*_0_(**r**) and a distribution of cell survival following the treatment *SF*(**r**), the probability of controlling the tumour is given by the following expression [18]:

(1) $$\begin{array}{c} \text{TCP}=\exp\left[-\overset{}{\underset{r}{\int }}n_{0}\left(r\right)\cdot SF\left(r\right)dr\right]. \end{array}$$

If the linear quadratic (LQ) model [30–32] adapted for proliferation [33] is used for describing the cellular survival, the fraction of cells surviving in a voxel **r** in the tumour is given by

(2) SFr=∏i=1nexp⁡−αr·dir−βr·di2r ·exp⁡Tln⁡2TDr,

where *n* is the number of fractions, *d*_*i*_(**r**) is the dose in fraction *i* in voxel **r**, *α*(**r**) and *β*(**r**) are the LQ parameters describing the radiosensitivity in voxel **r**, *T* is the treatment duration, and TD(**r**) is the cell doubling time in the voxel **r**. If the variation in radiation sensitivity is related to the oxygenation of the cells, *α*(**r**) and *β*(**r**) could be expressed as functions of the *α* and *β* parameters relevant for well oxygenated cells and the oxygen tension-dependent modification factors, OMF, and thus 2 becomes

(3) SFr=∏i=1nexp⁡−αOMFpO2r·dirmmmmkm−βOMF2pO2r·di2r ·exp⁡Tln⁡2TDr.

OMF (*pO*_2_(**r**)) is given by the following expression:

(4) $$\begin{array}{c} \text{OMF}\left(pO_{2}\left(r\right)\right)={\text{OMF}}_{\max}\frac{k+pO_{2}\left(r\right)}{k+{\text{OMF}}_{\max}pO_{2}\left(r\right)}, \end{array}$$

where OMF_max⁡_ is the maximum protection achieved in the absence of oxygen and *k* is a reaction constant as described by Alper and Howard-Flanders [34].

In these conditions, the probability for controlling the tumour could be written as

(5) TCP =exp⁡−∫rn0r·∏i=1nexp⁡−αOMFpO2r·dirmmmmmmmmmmmmkmmm−βOMF2pO2r·di2rimmmmm·exp⁡Tln⁡2TDrdr∏i=1nexp⁡−αOMFpO2r·dir.

Several proposals exist in the literature regarding the way the parameters *n*_0_(**r**), TD(**r**), and OMF(*pO*_2_(**r**)) could be derived from PET images. Thus, the density of the clonogenic cells in the beginning of the treatment could be derived from a FDG PET image based on the assumption that the enhanced FDG uptake should correspond to areas of higher density of glucose-avid clonogens [35]. As FLT is a marker taken up by the cells and phosphorylated by thymidine kinase 1 (TK1) which is an enzyme closely tied to cellular proliferation, it has been postulated that the retention of FLT within the cells provides a measure of cellular proliferation [36, 37]. The parameters used for describing the relationship between radiation sensitivity and tumour oxygenation could be derived based on the relative uptake of hypoxia-specific PET markers for various oxygen tensions, as proposed by Toma-Dasu et al. [38, 39], and subsequently tested with respect to its feasibility based on FMISO PET [19]. Thus, if FDG, FLT, and FMISO images of the tumour would be available before the start of the treatment and the relationships between the tracers uptake and *n*_0_(**r**), TD(**r**), and OMF(*pO*_2_(**r**)) would also be known, 5 could be used to determine the heterogeneous dose distribution that should be delivered for achieving a defined level of TCP.

Nevertheless, the practical implementation of this approach towards the complex BTV concept proposed by Ling et al. [1] is faced with several potential problems that may not be easily solved. Thus, one key problem is the fact that the required information about the biological parameters of interest would in fact be derived from PET images taken at different time points extending over several days to allow for the clearance of the different tracers. Therefore, assuming for example that the FDG PET image is taken at the time point *t*_1_ before the start of the treatment while FMISO and FLT images are taken at *t*_2_ and *t*_3_, respectively, 5 should be rewritten as

(6) TCP =exp⁡Tln⁡2TDr,t3−∫rn0r,t1mmmmmimm·∏i=1nexp⁡−αOMFpO2r,t2·dirmmmmmmmmimmkkm−βOMF2pO2r,t2·di2rmmmmmk·exp⁡Tln⁡2TDr,t3dr.

The expression in 6 shows that in reality it is quite difficult to speak about a time-independent BTV, given the spatial and temporal heterogeneity of the parameters determining the tumour control probability. Furthermore, the different biological processes that have to be incorporated are seldom coinciding in space [40, 41] meaning that several boost volumes would have to be defined, possibly with different boost levels.

The temporal stability and the reproducibility of the signal in the images before the start of the treatment given by the technical limitations of the imaging method, but, moreover, by the dynamics of the biological system that is imaged, may add yet another layer of complication to the already challenging task of multiparameter mapping of the target with respect to the radiobiological tumour features related to adverse response to (chemo)radiotherapy.

Last, but not least, the intrinsic radiosensitivity of the patients would have to be accounted for. Radiosensitivity parameters could be derived from clinical dose-response curves, but these are considered relevant for populations of patients which often exhibit considerable interpatient heterogeneity [42]. Instead one could use for example patient-specific parameters derived* in vitro* from biopsy materials as these were shown to better describe the response to treatment than generic or average parameters [43]. In the future such information may also be combined with predictive molecular assays providing quantitative information on the responsiveness of individuals to various forms of treatment. Therefore, the true individualisation of treatment would have to determine the right prescription levels not only for the BTV or its equivalents, but also for the CTV and the GTV.

## 4. Integrated Biological Dose Prescription and Treatment Adaptation Based on Functional Imaging: Is It Time for a New Paradigm Shift?

Given the inherent limitations in defining the BTV and determining the dose prescription that should overcome the radioresistent foci within the BTV, the implementation of simple dose painting approaches might lead to disappointing results in clinical settings. Therefore, the present paper proposes a paradigm shift from focusing on the radiobiological dose prescription towards biologically adapted radiation therapy, BIOART, based on tumour responsiveness assessed with functional imaging. The BIOART concept, in a much wider acceptation, was introduced by Brahme in 2003 as Biologically Optimized* in vivo* Predictive Assay-Based Adaptive Radiation Therapy [3]. The original paper proposed that combining accurate knowledge about the delivered dose acquired with a PET camera based on the nuclear reactions induced in the patient by ions or high energy photons, together with information regarding the density of tumour clonogens at some early point during the treatment derived from two successive PET images, one taken before the start of the treatment and one after about one week, could be used to assess the responsiveness of the tumour to the treatment and consequently to adapt the treatment. Although very appealing, the monitoring of the dose delivery or rather of the production of positron emitting isotopes inside the patient following nuclear reactions, in case of ion therapy, or photonuclear reactions in case of photon irradiation with high energy photons, is not yet possible as a routine clinical procedure [44, 45], the accurate dose determination being achieved at its best through deformable dose registration based on repeated CBCT images during the course of the treatment. The effectiveness of this approach has however been debated on the grounds of the associated uncertainties [46].

The second component of the generic BIOART approach, monitoring of the tumour response by repeated PET images early during the treatment, is actually currently feasible. Indeed, several studies have explored qualitatively the correlations between variations in PET tracer uptake and treatment outcome [47–53]. Nevertheless, a very recent study on NSCLC imaged with FDG-PET before the start of (chemo)radiotherapy and during the second week of radiation therapy showed that it is feasible to determine a threshold value for the effective radiosensitivity of the patients that could be used quantitatively to divide the patients into good and poor responders to treatment as assessed by overall survival at 2 years after treatment [54]. These results therefore support the high potential of early assessment of treatment responsiveness and subsequent treatment individualisation by identifying the likely candidates for more aggressive strategies like dose escalation or combined therapies needed to increase the rate of local control.

**Figure 1:**
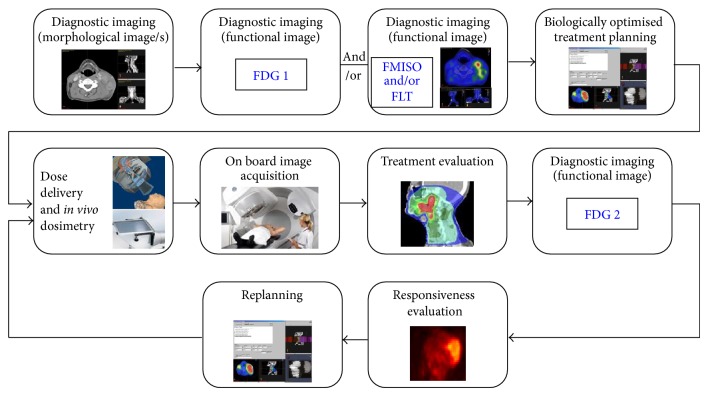
Schematic illustration of customised adaptive radiation therapy accounting for tumour hypoxia and/or proliferation.

This approach also provides another advantage, as repeated examinations in the same patient ensure that each patient is its own reference and does not require specific assumptions regarding the radiosensitivity. In fact, the approach opens the possibility of deriving effective radioresistance parameters that could eventually be used for treatment adaptation. Thus, assuming that the FDG images give a measure of the tumour burden, variations in signal intensities in individual voxels in the same patent would reflect changes in the density of functional clonogenic cells due to cell kill or proliferation. These and the dose distributions could then be used together with 2 or a simplified version of it to determine effective parameters for radiosensitivity [3, 54]. Other investigations of biologically adverse processes like hypoxia and proliferation could offer additional information that could be used for fine-tuning the parameters or included in the treatment optimisation process, provided that their intrinsic heterogeneity and dynamics are accounted for. A schematic illustration of the currently proposed approach for individualising the treatment and adapting it based on functional PET imaging is shown in Figure 1.

Several papers have shown that pretreatment FDG, FMISO, and FLT PET imaging might have not only prognostic values for indicating the likely course of the disease in the untreated individual, but also, more importantly in this setting, predictive values that might allow the selection of patients that would benefit from the chosen treatment [55–59]. However, proper patient selection and subsequent dose painting approaches for prescribing and delivering the dose do not guarantee the success of the treatment due to the large interpatient variability in response [56] and therefore they should be integrated in complex strategies for the management of the tumour which include early monitoring of the response and treatment adaptation.

Central to responsiveness evaluation and treatment adaptation is the registration of images containing anatomic, functional, and dosimetric information. This is the result of a mathematical optimisation process using algorithms aimed at aligning the images through rigid or deformable transformations [60]. Several sources of uncertainties exist in this process, some intrinsic to the algorithms used and other originating in uncertainties or noise in the analysed images. As there is the risk that these uncertainties might interfere with the analysis of the information in the images, it is important to include them in a sensitivity analysis aimed at testing the robustness of the results or predictions, as done, for example, by Tilly et al. [61]. It has to be highlighted that best results are probably obtained when the assessment of the response has to take place not later than two weeks from the start of the treatment. This not only prevents the inflammatory response from dominating the information that could be retrieved from repeated FDG images, but also minimises the morphological changes that may be caused, for example, by tumour shrinkage or progression and which might require deformable registration algorithms that are more error prone and therefore more of a source of uncertainties.

The proposal above has mainly been concerned with using functional information from PET images for treatment monitoring and adaptation. Nevertheless, similar consideration might also apply for functional magnetic resonance imaging (MRI) that allows both qualitative and quantitative characterisation of clinical tumours and the subsequent mapping of the tumour response. Thus, parameters derived from diffusion-weighted MRI (DW-MRI) could offer information on tissue cellularity and may therefore be used for treatment response monitoring [62]. Other methods like dynamic contrast-enhanced (DCE) imaging may be useful to obtain information on tissue vasculature [63], although it has been argued that tumour oxygenation might have a more complex dependence [64]. Proposals also exist for imaging lactate or choline levels in tumours as surrogates for hypoxia and proliferation [2].

## 5. Conclusions

There is no doubt that functional and molecular imaging have the potential to provide a paradigm shift in treatment planning and optimisation in cancer therapy that extends well beyond target definition. While multiparameter examinations will nevertheless provide valuable prognostic information for each patient, it is in unleashing the predictive power of the tracers that the true value of PET lies in modern radiotherapy. Thus, pretreatment investigations, possibly combined with predictive molecular information on the intrinsic features of each patient, will provide initial information on the dose levels needed to be included in the individual treatment plan and the likely therapeutic approaches. Subsequent examinations early during the treatment would then provide information on tumour responsiveness that may subsequently be used to determine the need for treatment adaptation taking into account the delivered dose distributions as well as adjuvant therapies, the effectiveness of which could also be assessed with this proposed approach. This may therefore be a simple and quite straightforward way to individualise treatment, considering not only the pretreatment condition of the patient, but also its intrinsic responsiveness and individual dose distributions that determine the need for later treatment adaptation. This is in fact the ultimate aim towards true individualisation of modern cancer treatment.
